# The Role of Verbal Auditory Hallucinations in Influencing and Retrospectively Predicting Physical Harm Prevalence in Early Psychosis

**DOI:** 10.2174/0117450179286452240520070533

**Published:** 2024-05-29

**Authors:** Cassie M. Hazell, Sophia Hasapopoulos, Jennifer McGowan, Roman Hamza, Zareena Ahmed, Ben Gaughan, Monica Huerga Malillos, Amber Gill, Amber Nomani, Emily Hickson, Anjeza Koruni, Faaisa Islam, Jonathan Souray, David Raune

**Affiliations:** 1 Department of Psychological Interventions, School of Psychology, University of Surrey, Guildford, GU2 7XH, UK; 2 Division of Psychology and Language Sciences, University College London, London, UK; 3 Harrow and Hillingdon Early Intervention in Psychosis (EIP) Service, London, UK

**Keywords:** Psychosis, First episode psychosis, Early intervention in psychosis, Physical harm, Violence, Quality improvement project

## Abstract

**Background:**

Research has established a relationship between psychosis and physical harm in the early course of psychosis. However, little is known about the relationship between specific psychosis symptoms, such as hearing voices, and physical harm.

**Objective:**

This study aimed to determine the prevalence and typology of physical harm related to hearing voices, as well as what aspects of the voice-hearing experience retrospectively predicted incidents of harm within an Early Intervention in Psychosis Service (EIPS).

**Methods:**

We conducted a quality improvement project in a single EIPS. We reviewed case notes of patients and extracted information on the cognitive-phenomenological features of the voices patients heard, as well as any incidents of physical harm that were causally linked to these voices.

**Results:**

It was found that 32.2% of EI patients had an actual incident of physical harm in their case notes that was causally linked to hearing voices. The most common type of physical harm was neglect. In terms of cognitive phenomenological binary correlations that retrospectively predicted physical harm in the case notes, patients were 20 and 7 times more likely to have harmed themselves if they heard self-harm commands (*i.e*., directions to harm themselves physically) and perceived the voice as omnipotent, respectively. Patients were 6 times more likely to have harmed someone else if they heard violent commands.

**Conclusion:**

Verbal auditory hallucinations commonly influence physical harm in the early course of psychosis. Hearing commands and/or believing the voice to be omnipotent are strong retrospective-correlative predictors that may aid in the assessment and therapeutic intervention.

## 1 INTRODUCTION

In the UK, early-course psychosis is defined as the three-year period following the first occurrence of delusions and/or hallucinations [1]. Hallucinations can be experienced in relation to any of the five senses, but the most frequently experienced are auditory hallucinations, also referred to as ‘hearing voices’ [2]. Hearing voices during the early course of psychosis is associated with poorer overall mental health and worse functioning [3]. Risk assessment is a core part of the care package offered to those under the care of Early Intervention in Psychosis Services (EIPS) [4]. These assessments focus on understanding the presence and future likelihood of any harm to or from the patient to themselves or those around them. This harm may be psychological, but the literature has largely focussed on physical harm in the context of psychosis. Physical harm in these studies is distinguished as being either physical harm to others (also referred to as violence) and/or physical harm to the self (*i.e*., self-harm and suicide).

A review of the literature reported that, on average, 34% of first-episode psychosis (FEP) patients had enacted some kind of violence towards others, with 16.6% being involved in a seriously violent act [5]. The risk of violence was greater for those with more severe symptomology [5]. For 1 in 10 of these patients, the violence warranted a criminal conviction [6]. However, once under the care of EIPS, the risk of future violence may be lower [7], as violent acts tend to occur before the diagnosis of FEP [8]. When trying to identify what aspects of psychosis are associated with violence, hearing distressing voices is considered to elevate the likelihood of actual violence [9]. A subtype of the voice-hearing experience, command hallucinations, has been identified as particularly risky [10], where these acts of violence represent compliance with their voices [11].

Physical harm to the self is also prevalent among those with early-course psychosis. Experiencing a first episode of psychosis can be a traumatic event in itself that can prompt suicidal ideation and attempts [12]. A review found that a pooled estimate of 18.4% of patients had engaged in self-harm prior to their FEP, with fewer (9.8%) cases of self-harming during the first year of their psychosis [13]. A similar prevalence of patients who had attempted suicide (21.6%) was reported [14], with one population cohort study finding a completed suicide rate of 4.3% in the early course of psychosis [15]. Depression is a common comorbid condition for those experiencing early-course psychosis [16], with as many as 80% of FEP patients reporting one or more periods of depression [17]. The presence of this low mood in the context of FEP can help explain the relationship between suicidality [18] or self-harm [19] and psychosis symptoms. Hearing voices in the context of psychosis is associated with increased suicidal thoughts and attempts [18] and self-harm [20]. Again, command hallucinations specifically have been implicated as elevating the risk of self-harm [21] and suicidality [22].

Hearing voices is a heterogenous experience. There are many sub-types of voices [23] that can vary in terms of their content, valence, and interpretation [24, 25]. Individual studies have, therefore, sought to go beyond the presence or absence of hearing voices as a predictor of physical harm and instead explore the relationship between specific dimensions of the voice-hearing experience and physical harm. Individual studies have reported that factors, such as violent content [26], familiarity with the voice [27], and beliefs that the voice is omnipotent [20], all elevate the risk of physical harm. However, to our knowledge, no study has combined the literature on voice-related risk factors and investigated their association with physical harm in a single study. Without this combined analysis, we are unable to determine whether these evidence-based risk factors are independent or do, in fact, explain shared variance.

Understanding violence, self-harm, and suicide in the context of early-course psychosis is of great importance, but it is not the only type of physical harm that patients may experience. For example, in routine clinical practice, we must assess and record any accidental injuries, harm from others, and self-neglect. Considering neglect, there is some suggestion in the literature that experiencing psychosis more broadly [28, 29], and early-course psychosis specifically [30], is associated with a decline in self-care, but far less is known about the relationship between self-neglect and specific psychosis symptoms, such as hearing voices. Extending the definition and typology of physical harm when assessing correlates of harm is important for current research to mirror risk assessment procedures in routine clinical practice.

The three areas that we suggest requiring further exploration are the need to move beyond past epidemiologically correlative research to explore the causal influence of voices, the relationship between a multitude of voice-related risk factors and physical harm in a single analysis (to isolate independent predictors), and for the conceptualisation of physical harm to be broadened to include wider domains. The data from this QIP has provided an opportunity to address these gaps in the literature. In the present paper, we bring together all of the voice-related risk factors that may be associated with physical harm into a single analysis to explore what factors predict multiple types of physical harm.

### 1.1 Aims

The aims of the present project are:

(1) To determine the prevalence of potential and actual incidents of physical harm in a single EIPS and the typology of this physical harm, where the health record indicates that voices were causal to the physical harm;

(2) To identify what evidence-based risk factors of the voice-hearing experience are associated with actual incidents of harm amongst patients with early-course psychosis.

## 2 MATERIALS AND METHODS

### 2.1 Design

The present study reports data from the Lived Experience Symptom Survey (LESS) project. LESS is a registered Quality Improvement Project (QIP) based on a single Early Intervention in Psychosis Service (EIPS) in the Central and North-West London (CNWL) Mental Health NHS Trust. The overall aim of the LESS project was to understand the presentation and assessment of hallucinations and delusions in an EIPS in order to inform service developments, such as improved multidisciplinary staff training on risk identification and management.

Our study used quantitative methods with both causal and correlative design components. The design permitted us to test the causal contributory influence of voice-hearing on physical harm incidents because the voice always occurred before the physical harm incident and there was a clear narrative link in patients’ case notes evidencing a causal link. The design relating to voice-related evidence-based risk factors was correlative because we do not know at what point after the onset of voices the evidence-based risk factor developed. We, therefore, use the term ‘prediction’ to refer to predicting the presence of physical harm in patients' case notes, but not whether or not the evidence-based risk factor preceded or caused the physical harm.

### 2.2 Participants

To be eligible for inclusion in the LESS project, patients were under the care of an EIPS based in CNWL at the time of the project. Patients may have been under the care of or received treatment from other mental health services previously. All EIPS patients were included in the project; however, this paper only reports the data of patients who reported experiencing auditory hallucinations as part of their psychosis presentation.

### 2.3 Lived Experience Symptom Survey (LESS)

The LESS was developed by the project lead (DR) specifically for the purposes of this QIP. The LESS form was designed to collect comprehensive information about the different cognitive-phenomenological features of a patient’s auditory hallucinations. Isaacson *et al*. (2022) provided further information on the LESS form. Within the LESS QIP, assistant psychologists reviewed the case notes of all patients within the EIPS. They extracted all information about their voice-hearing experiences and inputted this into the LESS form. The assistant psychologists received training from the team of clinical psychologists on the LESS form and auditory hallucinations. The data was anonymised at the point of extraction. The information on the LESS forms is the data source for the current study.

### 2.4 Measures

We collected basic demographic and clinical data from all participants to describe the sample. This information was taken from their most recent case note.

#### 2.4.1 Evidence-Based Risk Factors (EBRFs)

We conducted a review of the literature using a keyword search of the Medline and PsycINFO databases. The aim of the review was to identify any risk factors that were found to be associated with physical harm in the context of auditory hallucinations. Risk factors were included, on which at least one empirical study found confirmatory evidence of the link between that factor and voice-related physical harm. From these papers, we identified a total of 12 hallucination-related evidence-based risk factors (EBRFs) that have previously been found to be associated with actual or potential physical harm (Table **1**). The full list of studies reviewed to identify the EBRFs is included in the Appendix.

#### 2.4.2 Physical Harm

Physical harm is defined as any act or event that results in clinically significant physical damage and/or injury. We assessed physical harm in two ways: firstly, assessing whether it was present or not, and secondly the typology of this harm. We assessed whether physical harm was present or not, and if present, whether it had occurred (actual harm) or if clinicians perceived it was likely to occur in the future (potential harm). We also developed a typology of physical harm based on definitions of physical abuse and neglect [31, 32] and assessments of physical harm used within our routine clinical practice. Five types of physical harm were identified: (1) harm to self – an intentional act that harms the patient themselves, (2) harm to others – an intentional act from the patient that harms someone else, (3) harm from others – any incident where the patient is harmed by someone else, (4) accidents – an action or incident where the patient is harmed but did not mean to harm themselves (*e.g*., risky behaviours where they could get injured), and (5) neglect – where a patient is harmed due to a lack of self-care. These categories reflect differences in the recipient of the harm and the intention behind it. Only incidents of physical harm that were related to voice-hearing experiences were included. That is, “I did X because of Y” – where X is some kind of physically harmful act, and Y is some aspect of their voice-hearing experience.

### 2.5 Procedure

APs working in the same EIPS reviewed the LESS forms that were completed based on the case notes of patients. The LESS forms were firstly rated in terms of whether each EBRF was present (scored as 1) or absent (scored as 0). An EBRF was coded as ‘absent’ if a factor was either not recorded at all or was explicitly recorded as not being relevant for that patient. Secondly, the LESS forms were scored in terms of whether physical harm was present or absent and, if present, its typology. To illustrate, a case note of “patient used a pair of scissors to cut her thigh saying it was due to hearing the voice” (fictional example) would be recorded as an ‘actual harm’, and an example of ‘harm to self’.

### 2.6 Analysis Plan

To determine the prevalence of physical harm, we calculated the frequencies and percentages of patients who showed at least one incident of physical harm and the typology of the incident. We did not count the total number of incidents per patient. Instead, we coded either the presence (1) or absence (0) of physical harm in case notes where it was causally linked to voices, so each patient could only score 1 or 0. To identify which EBRFs retrospectively predicted the presence of actual physical harm causally related to voices, we conducted a series of logistic regressions. The predictors in all models were the EBRFs, and the dependent variables were the presence of physical harm (either present or absent) to the self or to others in separate regression models. In line with the recommendations of Jenkins and Quintana-Ascencio [33], logistic regressions were run only where a minimum of 8 observations were recorded for each of the predictor and outcome variables. Where 8 or more observations were present, the EBRFs included in the regression models were limited to the factors that were found to be linked to that particular type of physical harm in the literature. The a priori regression models were therefore planned as follows: (1) actual harm to self: CH – self-harm content, CH – violent content to others, CH – non-specific harmful content, CH – familiar voice identity, CH – a voice not female, omnipotence, malevolence, congruent delusions, derogatory comments, and threatening comments; and (2) actual harm to others: CH – violent content, CH – non-specific harmful content, CH – familiar voice identity, CH – voice not female, omnipotence, congruent delusions, and derogatory comments. The size of the effect of each logistic regression was calculated using odds ratios (*i.e*., the exponential value of B (Exp(B))). An Exp(B) greater than one indicates an increased chance of the event occurring.

### 2.7 Ethics

The data presented here was collected as part of a registered NHS QIP, and therefore, ethical approval was not sought or required. The data was handled in line with the General Data Protection Regulations (GDPR) and NHS data security policies and procedures.

## 3 RESULTS

### 3.1 Sample Characteristics

The data from 208 EIPS patients were included in this project. The patients were mostly young males with a schizophrenia spectrum diagnosis. The average duration of psychosis symptoms was just over two years, and the average duration of untreated psychosis was just over two months. Table **2** presents a summary of the sample characteristics.

### 3.2 Prevalence of Physical Harm

Almost a third of patients had some kind of actual physical harm recorded in their case notes. The most common type of physical harm was neglect, followed by harm to self. The potential for future harm was noted for 22.6% of patients. The exact frequencies are mentioned in Table **3**.

### 3.3 Prevalence of Evidence-based Risk Factors (EBRFs)

The most frequently reported EBRFs were the presence of congruent delusions and voices that were malevolent and/or made derogatory comments. These factors were found in more than 30% of patients' case notes. More than a quarter of patients heard voices that were perceived as omnipotent and/or contained commands to harm themselves. The EBRF found least often was the combined experience of voices being threatening and omnipotent, and also the voice making benevolent commands. The exact frequencies are mentioned in Table **3**.

### 3.4 Excluded Variables

The following variables were not included in any logistic regressions as less than 8 observations were recorded: (1) EBRFs: (a) CH – benevolent content belief (*n*=2), and (b) CH – threatening and omnipotence belief (*n*=2); and (2) physical harm: (a) potential harm from others (*n*=4), (b) potential neglect (*n*=0), (c) actual harm from others (*n*=1), and (d) actual accident (*n*=2).

### 3.5 Retrospective Prediction of Actual Harm

We first tested whether any of the EBRFs predicted the presence of actual physical harm to the patient themselves. A significant model of EBRFs predicting actual harm to self was found, explaining 47.0% of the model variance (*χ2* (10) = 63.79, *p* < .001). We found two significant predictors, specifically hearing hallucinations that command the hearer to engage in self-harm (Wald *χ2* (10) = 21.39, *p* < 0.001) and beliefs that the voice is omnipotent (Wald *χ2* (10) = 11.69, *p* < 0.001). Patients were more than 20 times more likely to harm themselves if they heard self-harm-related commands than those who did not, and 7 times more likely if their voice was perceived as omnipotent.

We also found a significant model of the EBRFs predicting actual harm to others that explained 17.5% of the total model variance (*χ2* (10) = 15.86, *p* = 0.03). Hearing a voice that makes violent commands was the only significant predictor in this model (Wald χ2 (7) = 8.10, *p* = 0.004). Those who heard violent command hallucinations were almost 6 times more likely to actually harm someone else than those who did not. The exact values are mentioned in Table **4**.

## 4 DISCUSSION

The main aim of the LESS QIP was to provide information about patients’ delusions and hallucinations that would aid in the improvement of multidisciplinary staff training about physical harm risk identification and management in our patient caseload. Using the data taken from patients’ case notes from a single EIPS, we determined the prevalence of physical harm that was causally related to voices amongst patients. We also explored what aspects of the voice-hearing experience retrospectively predicted incidents of actual harm. It was found that more than half of patients had either an actual or potentially physically harmful incident recorded in the case notes, with a third involved in some form of actual physical harm. Neglect was the most common type of physical harm, followed by harm to self. Patients were 20 times more likely to harm themselves if they heard self-harm commands and 7 times more likely if they perceived their voice as omnipotent. Patients were 6 times more likely to harm someone else if they heard violent commands.

The rates of actual physical harm towards others (7.7%) and the self (14.4%) were lower than those found in previous studies, which ranged from 16.6% to 34.5% [5, 13, 14]. All of the patients included in this project were under the care of EIPS. The reduced rates of harm may, therefore, reflect the overall reduction in physical harm seen when patients come under the care of EIPS [7]. Alternatively, the differing results could be attributed to only coding physical harm that was linked to the voice-hearing experience as opposed to any physical harm that occurred in the context of FEP. Even though the prevalence of harm was lower amongst the patients included in our project, the figures are highly clinically significant. Our findings support the conclusions of previous studies that physical harm may be a common part of early-course psychosis in patients.

The most common type of physical harm recorded in case notes of patients was neglect. Historically, self-neglect was considered to be an indicator of imminent relapse among patients with psychosis [34], but more recently, it has been recognised as a symptom of early course [35] and chronic psychosis [36]. There is little available evidence on the prevalence of self-neglect amongst patient cohorts. This may be in part due to the difficulties healthcare professionals experience in identifying self-neglect as they balance their duty of care with respecting patients’ autonomy [37]. Our data shows that self-neglect is common amongst FEP patients, with at least 1 in 5 having evidence of actual neglect in their case notes. However, further research is needed to determine whether the high prevalence of neglect found is replicated in other EIPS.

Command hallucinations that instruct the patient to harm themselves or others substantially (21 times) increased the risk of harm to the self and others, respectively. The link between physical harm and command hallucinations is well documented in the literature. The association between command halluci- nations and violence is not isolated to EIPS but is evident within inpatient [38] and forensic [39] settings as well. There is also robust evidence of the link between self-harm commands and self-injurious behaviour [21]. However, not all patients who hear command hallucinations act upon them. Our study is not able to offer any definitive explanations for why some patients do and do not comply with their voices. Potential explanations for compliance include the content of the command, beliefs about the voice, and beliefs about the consequences of non-compliance [11]. In their cognitive model of compliance, Beck-Sander *et al*. [40] formulated that acting upon commands is the result of an interaction between beliefs about the voice and the command content, with overall compliance less likely in those who do not perceive their voice as omnipotent and higher amongst certain command sub-types where voices are perceived as benevolent. While our findings support that the mere presence of command hallucinations is a strong risk factor for physical harm, the precision of this odds ratio, and therein the accuracy that clinicians can identify those patients at great risk of harming themselves or others, could be enhanced by considering factors related to compliance.

We found that patients who believed their voice(s) was omnipotent were more likely (almost 6 times) to harm themselves. Omnipotence is a key construct within the cognitive-behavioural model of voice-hearing (CBTv) [24]. Believing that the voice is all-powerful is synonymous with the hearer feeling they have no control and are disempowered. Omnipotence beliefs have consistently been found to predict compliance with harm-related command hallucinations [41]. However, there is mixed evidence as to the relationship between omnipotence beliefs and harm related to the self specifically (some studies have found an association [42], while others have not [20]). Our findings support the adverse effects of perceiving the voice(s) as omnipotent [42] and suggest the importance of assessing such beliefs when assessing patients’ voice-hearing experience [31]. Other voice-related beliefs included in the CBTv model were also tested, but they were not significantly associated with any type of physical harm. This may be because beliefs related to the voice's intent and omniscience do not explain any unique or additional variance to that explained by the other EBRFs. Alternatively, it may be that such beliefs predict some of the other aforementioned adverse outcomes, such as distress [43], but are not associated with physical harm. However, we could not use the data collected to determine which of these explanations is more probable.

### 4.1 Limitations

The findings from this paper reflect an advancement in our understanding of physical harm amongst patients with early-course psychosis and what voice-related EBRFs retrospectively predicted actual incidents of such harm. Our findings go beyond mere associations; as for incidents of physical harm to be included in the present analysis, there must be a narrative link between hearing a voice and physical harm, thus suggesting a direction in this relationship. However, our findings cannot determine whether there is a causal relationship between the EBRFs and physical harm, nor can we make any claims as to whether a dose-response relationship exists. We did not record the frequency or severity of physically harmful incidents or the number of EBRFs present for a single patient. We, therefore, cannot determine whether any of the EBRFs included predict the frequency or severity of harm or whether there is a causally additive effect of EBRFs on harm outcomes. Also, our limited resources meant that while we were able to collect data from 208 patients, we did not know how many other patients had not yet been assessed during a period of short-staffing. This non-consecutive methodology represents a representativeness limitation but does not involve any systematic bias.

This study focuses on the relationship between voice-related EBRFs and physical harm. Moreover, the independent variance explained (47% for self-harm and 17.5% for harm to others) indicates that there are likely to be other causal influences not measured in the present study. The literature indicates that there are a number of other factors that may increase or decrease a person’s likelihood of enacting or being involved in a harmful incident. For example, the content of unusual beliefs (also referred to as delusions) has been found to predict violent behaviour [9, 44] but may be unrelated to suicidal ideation [45]. However, further research is needed to identify whether such other factors explain unique or shared variance when predicting the occurrence of physical harm.

The present project makes use of data from the LESS QIP based on a single EIPS. Being a QIP rather than a research study can mean that our methodology is susceptible to bias. For example, we did not assess the inter-rater reliability of the data extraction from patient case notes. Also, the findings may not generalise to other services. Using case notes of patients as the original data source could present issues for the validity of our findings, as our results are dependent on the quality of these case notes. The comprehensiveness of such records can be variable [31], but we are confident that the prevalence and typology of the physical harm reported here are accurate, as recording such information is a priority for mental health services [46].

A future study could use semi-structured face-to-face research interviews with patients to separate the voice features and overcome any possible case note limitations. Furthermore, a key limitation of our study is that we were not able to date the physical harm and the development of specific evidence-based cognitive features. Therefore, the high odds ratios found could theoretically be additionally influenced by a bidirectional effect of cognitive features influencing physical harm, but also patients who are physically harmed might then have an increased likelihood of developing the cognitive features. For example, patients who have harmed themselves or others might theoretically then start hearing commands to do it. So, the high retrospective correlative odds ratios found should be interpreted with case note limitation and by keeping theoretical bidirectionality in mind.

### 4.2 Research Implications

Our findings require replication in other settings as part of a purposive research study. Some potential additional research questions building on this work include: what other factors outside of those related to hearing voices predict physical harm? Do these factors represent novel risk factors? A priority for future research is to test whether any of the EBRF associations found here are causal. However, experimental tests that involve physical harm as an outcome can be difficult to conduct for safeguarding reasons. Instead, intervention trials offer an ethically sound approach to test for causality. The COMMAND trial, for example, aimed to reduce harmful compliance with voices *via* cognitive therapy [47]. The underlying theory of the therapeutic model is that harmful compliance is a product of believing that the voice is omnipotent, omniscient, and malevolent [48]. As such, the post-therapy effectiveness of the COMMAND intervention supports a causal relationship between the afore- mentioned beliefs about voices and physical harm.

One issue with studies, such as the COMMAND trial and cognitive behaviour therapies for psychosis (CBTp) more broadly [49], is that they tend to target multiple mechanisms simultaneously, making it difficult to ascertain which target(s) is causing the adverse outcome. To illustrate, the effects of COMMAND may be driven by changes in all three beliefs (*i.e*., improvements in omnipotence, omniscience, and malevolence) or only one or two of them. However, because these beliefs are all targeted in the same intervention, we cannot isolate their effects. The development of streamlined and focused interventions that target a single mechanism will enable us to explore the causal relationship between hearing voices and physical harm and enhance the effectiveness of psychological interventions for voice hearing.

### 4.3 Clinical Implications

Assuming our findings generalise to other EIPSs, we have identified that substantial numbers of voice hearers experience physical harm, at least partly due to hearing the voices. We have also identified several voice-related EBRFs that increase the likelihood of physical harm being present in the case notes of patients experiencing early-course psychosis. A recent review indicates that some of the EBRFs (*i.e*., self-harm command hallucinations) are currently neglected when assessing the risk of physical harm amongst psychosis patients [50]. We suggest that the presence of these EBRFs should help in risk assessments and provide an indicator that someone may be more likely to harm themselves and/or others. However, it is important to acknowledge that these are ‘risk’ factors, not determinants. Patients reporting that their voice is making violent and/or self-harm commands do have an increased risk of physical harm, but it is not an inevitability. Equally, it is possible that patients may physically harm themselves or others without hearing these types of command hallucinations or holding omnipotent beliefs about the voice.

## CONCLUSION

In conclusion, the present project provides evidence for the high prevalence of physical harm in early-course psychosis influenced by verbal auditory hallucinations. We found that the risk of physical harm was greatest amongst those patients who heard command hallucinations and/or perceived the voice as omnipotent. Clinicians may find it helpful to consider which of their service users may be at greatest risk of physical harm based on aspects of their voice hearing experience, alongside other indicators of risk. However, importantly, these risk factors are not conclusive or exhaustive. Further research is needed to replicate these findings in other clinical settings and test if the retrospectively correlated features will prospectively predict and causally influence physical harm.

## AUTHORS' CONTRIBUTIONS

It is hereby acknowledged that all authors have accepted responsibility for the manuscript's content and consented to itssubmission. They have meticulously reviewed all results and unanimously approved the final version of the manuscript.

## Figures and Tables

**Appendix:** Supporting evidence for each EBRF.

**EBRF**	**Evidence**	** N **	**Design**	**Type of Harm**
** CH – self harm content **	-	-	-	-
	(Fox *et al.*, 2004)	32	Cross-sectional	Self-Harm
	(Lee *et al.*, 2004)	53	Cross-sectional	Self-Harm
	(Rogers *et al.*, 2002)	100	Retrospective cohort	Self-Harm, Suicide
** CH – violent content **	-	-	-	-
	(Nielssen *et al.*, 2007)	88	Retrospective cohort	Homicide
	(Lee *et al.*, 2004)	53	Cross-sectional	Violence, Self-Harm
	(Fox *et al.*, 2004)	32	Cross-sectional	Violence
** CH – non-specific harmful content **	-	-	-	-
	(Wong *et al.*, 2013)	148	Retrospective cohort	Suicidal Ideation, Suicide Attempt
	(Yee *et al.*, 2011)	74	Retrospective cohort	Assault
	(Fedyszyn *et al.*, 2011)	607	Retrospective cohort	Suicide Attempt
	(Junginger, 1990)	51	Cross-sectional	Suicide Attempt, Self-Harm, Violence
** CH – benevolent content **	-	-	-	-
	(Fox *et al.*, 2004)	32	Cross-sectional	Self-Harm, Violence
	(Beck-Sander *et al.*, 1997)	35	Cross-sectional	Violence
** CH – familiar voice identity **	-	-	-	-
	(Junginger, 1990)	51	Cross-sectional	Suicide Attempt, Self-Harm, Violence
** CH – threatening and omnipotence **	-	-	-	-
	(Shawyer *et al.*, 2008)	75	Cross-sectional	Self-Harm, Violence
** CH – voice not female **	-	-	-	-
	(Shawyer *et al.*, 2008)	75	Cross-sectional	Self-Harm, Violence
** Omnipotence **	-	-	-	-
	(Connor & Birchwood, 2013)	74	Cross-sectional	Suicidal Ideation
	(Simms *et al.*, 2007)	33	Cross-sectional	Suicidal Intent of Self-Harm
	(Peters *et al.*, 2012)	46	Cross-sectional	Suicidal Ideation
	(Stratton *et al.*, 2017)	25	Cross-sectional	Homicide
	(Bucci *et al.*, 2013)	32	Cross-sectional	Violence, Self-Harm, Suicide Attempt
	(Fox *et al.*, 2004)	32	Cross-sectional	Self-Harm, Violence
	(Beck-Sander *et al.*, 1997)	35	Cross-sectional	Violence
** Malevolence **	-	-	-	-
	(Simms *et al.*, 2007)	33	Cross-sectional	Self-Harm, Suicidal Intent of Self-Harm
	(Peters *et al.*, 2012)	46	Cross-sectional	Suicidal Ideation
** Congruent delusions **	-	-	-	-
	(Nielssen *et al.*, 2007)	88	Cohort	Homicide
	(Shawyer *et al.*, 2008)	75	Cross-sectional	Violence
	(Beck-Sander *et al.*, 1997)	35	Cross-sectional	Violence, Self-Harm
** Derogatory comments **	-	-	-	-
	(Sheaves, Johns, Griffith, Isham, Kabir, *et al.*, 2020)	15	Grounded theory	Self-Harm, Suicidal Ideation, Suicide Attempt
	(Cheung *et al.*, 1997)	62	Cross-sectional	Violence
** Threatening comments **	-	-	-	-
	(Sheaves, Johns, Griffith, Isham, Kabir, *et al.*, 2020)	15	Grounded theory	Self-Harm, Suicidal Ideation, Suicide Attempt

**Note:** CH = command hallucination(s); EBRF = evidence-based risk factor.

**Table 1:** EBRFs associated with physical harm in psychosis with descriptions.

**EBRF**	**Example Evidence/Refs**	**Explanation**
**Content of Voice**	-	-
CH – self-harm content	[21]	Hallucination(s) that commands the patient physically harms themselves.
CH – violent content to others	[26]	Hallucination(s) where the voice commands physical harm using a violent method.
CH – non-specific harmful content	[51]	Hallucination(s) where the voice commands physical harm using a non-violent method.
CH – a voice not female	[52]	The command hallucination(s) content is a non-female voice.
Derogatory comments	[9]	The hallucination(s) makes comments that are critical of the patient.
Threatening comments	[53]	The hallucination(s) makes comments that the voice intends to cause the patient harm that may or may not be physical.
CH – familiar voice identity	[27]	The command hallucination(s) content is someone known to the patient.
**Content of Voice *plus* Belief**	-	-
CH – threatening and omnipotence	[52]	The command hallucination(s) contains threatening material and is perceived as omnipotent.
Congruent delusions	[26]	The patient experiences delusional beliefs where the content is aligned with hallucination(s) content.
**Beliefs about Voices**	-	-
Malevolence	[42]	The hallucination(s) is believed to have negative intentions towards the patient.
Omnipotence	[20]	The hallucination(s) is believed to be all powerful.
CH – benevolent content	[40]	Hallucination(s) where the voice is perceived to command physical harm as a benevolent act.

**Note: **CH = command hallucination(s); EBRF = evidence-based risk factor.

**Table 2:** Sample characteristics.

**Variable**	**M(SD) or N(%)**
Age (years)	25.2 (5)
Psychosis length (months)	28.8 (17)
Duration of untreated psychosis (months)	2.3 (5.9)
Gender	-
Male	150 (72.1)
Female	58 (27.9)
Ethnicity	-
Unknown	12 (5.8)
White	41 (19.7)
Black	50 (24)
Asian	75 (36.1)
Other	30 (14.4)
Religion	-
Unknown	78 (37.5)
Christian	31 (14.9)
Islam	53 (25.5)
Hindu	18 (8.7)
Buddhism	1 (0.5)
Not Religious	19 (9.1)
Other	8 (3.8)
Diagnosis	-
Schizophrenia Spectrum	84 (40.4)
Affective Psychosis	29 (13.9)
Acute and Transient	56 (26.9)
Delusional Disorder	2 (1)
Substance-Induced Psychosis	4 (1.9)
Psychosis NOS	28 (13.5)
Other	5 (2.4)

**Note: **M = mean; SD = standard deviation; Age = age at the time of LESS data collection; Psychosis length = the time between first episode of psychosis and LESS data collection; Duration of untreated psychosis = the time between first episode of psychosis and first dose of antipsychotic medication.

**Table 3:** The prevalence of physical harm and evidence-based risk factors (EBRFs) amongst EIPS patients.

-	**Actual Harm Present** **n(%)**	**EBRF Present** **n(%)**	**Potential Harm** **n(%)**	**No Harm** **or** **EBRF Absent** **n(%)**
**Physical harm**	-	-	-	-
Overall	67(32.2)	-	47(22.6)	94(45.2)
Harm to self	30(14.4)	-	26(12.5)	152(73.1)
Harm to others	16(7.7)	-	21(10.1)	171(82.2)
Harm from others	1(0.5)	-	4(1.9)	203(97.6)
Accident	2(1.0)	-	8(3.8)	198(95.2)
Neglect	47(22.6)	-	0(0)	161(77.4)
**EBRFs**	-	-	-	-
CH – self-harm content	-	56(26.9)	-	152(73.1)
CH – violent content	-	34(16.3)	-	174(83.7)
CH – non-specific harmful content	-	18(8.7)	-	190(91.3)
CH – benevolent content	-	2(1.0)	-	206(99.0)
CH – familiar voice identity	-	9(4.3)	-	199(95.7)
CH – threatening and omnipotence	-	2(1.0)	-	206(99.0)
CH – voice not female	-	20(9.6)	-	188(90.4)
Omnipotence	-	56(26.9)	-	152(73.1)
Malevolence	-	63(30.3)	-	145(69.7)
Congruent delusions	-	63(30.3)	-	145(69.7)
Derogatory comments	-	71(34.1)	-	137(65.9)
Threatening comments	-	48(23.1)	-	160(76.9)

**Note: **n = frequency; CH = command hallucinations.

**Table 4:** Logistic regression of evidence-based risk factors predicting the presence and typology of physical harm.

-	***χ2***	***R^2^***	**Wald *χ2***	***p***	**Exp(B)**
**Actual harm to self**	63.79	47.0%	-	<.001	-
-	9	%	-	-	-
CH – self-harm content*	-	-	21.39	<.001	20.20
Omnipotence*	-	-	11.69	<.001	7.32
CH – non-specific harmful content	-	-	0.11	.74	1.30
Threatening comments	-	-	1.40	.24	0.46
Derogatory comments	-	-	2.84	.09	0.34
Malevolence	-	-	1.11	.29	1.95
CH – violent content	-	-	.37	.55	0.68
CH – familiar voice identity	-	-	0.59	.44	0.42
CH – voice not female	-	-	3.50	.06	3.87
Congruent delusions	-	-	1.05	.31	0.54
**Actual harm to others**	15.86	17.5%	-	.03	-
CH – violent content*	-	-	8.10	.004	5.93
CH – non-specific harmful content	-	-	0.00	.97	1.03
Derogatory comments	-	-	1.10	.29	1.87
Omnipotence	-	-	0.12	.72	1.24
CH – familiar voice identity	-	-	3.63	.06	5.35
CH – voice not female	-	-	0.85	.36	0.45
Congruent delusions	-	-	0.01	.93	0.94

**Note:** *χ2* = model summary statistic; Wald *χ2*= individual predictor statistic; CH = command hallucinations; * *p* < .05..

## Data Availability

The anonymised data file is available upon request from the corresponding author [C.H].

## ABBREVIATIONS

CH: Command hallucination(s)
EBRF: Evidence-based risk factor
